# Hyperhomocysteinemia Associated with Low Muscle Mass, Muscle Function in Elderly Hemodialysis Patients: An Analysis of Multiple Dialysis Centers

**DOI:** 10.1155/2019/9276097

**Published:** 2019-06-09

**Authors:** Chi-Sin Wang, Te-Chih Wong, Tuyen Van Duong, Chien-Tien Su, Hsi-Hsien Chen, Tso-Hsiao Chen, Yung-Ho Hsu, Sheng-Jeng Peng, Ko-Lin Kuo, Hsiang-Chung Liu, En-Tzu Lin, Yi-Wei Feng, Shwu-Huey Yang

**Affiliations:** ^1^School of Nutrition and Health Sciences, Taipei Medical University, Taipei, Taiwan; ^2^Department of Nutrition and Health Sciences, Chinese Culture University, Taipei, Taiwan; ^3^School of Public Health, Taipei Medical University, Taipei, Taiwan; ^4^Department of Family Medicine, Taipei Medical University Hospital, Taipei, Taiwan; ^5^Division of Nephrology, Department of Internal Medicine, School of Medicine, College of Medicine, Taipei Medical University, Taipei, Taiwan; ^6^Division of Nephrology, Department of Internal Medicine, Taipei Medical University Hospital, Taipei, Taiwan; ^7^Department of Nephrology, Taipei Medical University-Wan Fang Hospital, Taipei, Taiwan; ^8^Division of Nephrology, Department of Internal Medicine, Taipei Medical University-Shuang Ho Hospital, Taipei, Taiwan; ^9^Division of Nephrology, Cathay General Hospital, Taipei, Taiwan; ^10^Division of Nephrology, Taipei Tzu-Chi Hospital, Taipei, Taiwan; ^11^Department of Nephrology, Wei Gong Memorial Hospital, Miaoli, Taiwan; ^12^Department of Nephrology, Lotung Poh-Ai Hospital, Yilan, Taiwan; ^13^Research Center of Geriatric Nutrition, Taipei Medical University, Taipei, Taiwan; ^14^Nutrition Research Center, Taipei Medical University Hospital, Taipei, Taiwan

## Abstract

**Background:**

The hyperhomocysteinemia was with high prevalence and has been considered as a risk factor for cardiovascular disease in hemodialysis patients. These patients also experienced a high risk of muscle wasting caused by the comorbidity, malnutrition, and low physical activity. We investigated the associations of homocysteinemia with muscle mass, muscle function in elderly hemodialysis patients.

**Methods:**

A clinical cross-sectional study was conducted on 138 hemodialysis patients aged 65 years and above in seven hospital-based hemodialysis centers in Taiwan. The data on anthropometry, laboratory, and 3-day dietary intake was examined. The skeletal muscle mass (SMM) was measured by the bioelectrical impedance analysis; the SMM was adjusted by height or weight as SMM_Ht2_ (kg/m^2^) and SMM_Wt_ (%). Muscle function was defined as handgrip strength (HGS) (kg) measured by handgrip dynamometer. Statistical analyses were conducted using simple regression and multivariable stepwise regression analysis.

**Results:**

In the total sample, 74.6 % of hemodialysis patients were hyperhomocysteinemia (≥ 15 *μ*mol/L). The means of SMM_Ht2_, SMM_Wt_, arm lean mass, hand grip strength, and muscle quality were 8.7 ± 1.2, 37.7 ± 5.6, 1.7 ± 0.5, 21.1 ± 7.4, and 10.0 ± 3.0, respectively. The multivariable stepwise regression analysis showed that homocysteinemia level was significantly inversely associated with SMM_Wt_ (B-coeff. = -0.03,* p *= 0.02) in hemodialysis patients above 65 years old, but not with muscle function.

**Conclusions:**

Hyperhomocysteinemia is common and associated with decreased muscle mass in the elderly hemodialysis patients. Future studies are suggested to explore the impact of the homocysteine-lowering therapy on muscle decline.

## 1. Introduction

The United States Renal Data System reported that the prevalence of treated end-stage renal disease (ESRD) was highest for individuals aged 65-74 years in Taiwan [1]. The elderly hemodialysis patients experienced the high risk for muscle wasting, which was related to higher morbidity and mortality [2]. Muscle wasting was the most common condition in elderly people, especially those undergoing hemodialysis with a high prevalence of decreased muscle mass and muscle function, varying from 12.7% to 45.1% in hemodialysis patients [3], the declines in muscle mass and function caused by age [3], comorbidity like diabetes mellitus and infections [4], malnutrition [5], and physical inactivity [6]. In addition, physicians prescribed exercise restrictions in those patients with complications during the treatment [7]. Furthermore, declines of muscle mass and function were exaggerated due to the long bedridden time for the dialysis, low physical function [8], low exercise capacity [9], and increased muscle atrophy [6].

The hyperhomocysteinemia was presented in 80% to 90% of hemodialysis cases [10]. Homocysteine (Hcy) has been considered an important cardiovascular risk factor [11]. Moreover, studies have established an association between high levels of homocysteine and a decline in physical function in elderly populations [12]. The lower muscle mass was observed in higher plasma homocysteine group in ≥ 65 years adults [13]. The effects of hyperhomocysteinemia in vascular and myocyte function leading to impaired muscle function were summarized as follows: (i) oxidative defense reduced and production of reactive oxygen species enhanced, (ii) inhibition of nitric oxide (NO) signaling, (iii) inflammation and its associated changes, and (vi) endoplasmic reticulum (ER) stress enhanced [14]. Previous empirical studies have shown elevated Hcy associated with the decline of muscle strength and physical function in the older adults [15, 16].

Since hemodialysis patients are with a high prevalence of hyperhomocysteinemia and in a high-risk group of muscle wasting, however, there are a limited number of studies that discussed this issue in hemodialysis patients, especially in the elderly. Therefore, we aim to investigate the association between homocysteine level and muscle mass, muscle function in elderly hemodialysis patients.

## 2. Materials and Methods

### 2.1. Study Design and Patients

A clinical cross-sectional design was conducted from September 2013 to April 2017 in seven hospital-based hemodialysis centers in Taiwan. The study was approved by the Taipei Medical University Joint Institutional Review Board (no. TMU-JIRB 201302024) for conducting in Taipei Medical University Hospital, Taipei Medical University-Wan Fang Hospital, Taipei Medical University-Shuang Ho Hospital, Wei Gong Memorial Hospital, and Lotung Poh-Ai Hospital, the institutional Ethics Committee from Cathay General Hospital (no. CGH-OP104001), and Taipei Tzu-Chi Hospital (no. 04-M11-090). All patients signed written informed consent forms before their participation.

The total sample of 138 patients aged 65 years and above and undergoing hemodialysis was recruited for the study. Patients who received stable hemodialysis treatment in the previous 3 months with equilibrated Kt/V of 1.2 and higher were included. Patients with obvious edema, hyperthyroidism, hypothyroidism, amputation, malign tumor, pregnancy, or hospitalization for renal disease reason were excluded.

### 2.2. Data Collection

#### 2.2.1. Demographics Data

We conducted chart reviews to collect the data related to age, gender, dialysis vintage, diabetes mellitus (DM), hypertension, cardiovascular disease (CVD), and the anthropometry data including dry weight, height, and interdialytic weight gain.

#### 2.2.2. Physical Activity

Patients' physical activity was assessed using the short version of the International Physical Activity Questionnaire (IPAQ) [17]. Interviewers recorded the average number of days per week and the average time per day that patient spent on exercising (vigorous, moderate, or walking exercise) in the past 7 days. The value of metabolic equivalent (MET in kcal/day) value was used to examine the levels of physical activity [17].

#### 2.2.3. Dietary Intake Data

All patients wrote down a three-day dietary record, including one dialysis day, one nondialysis day, and one day during the weekend. The data collection of dietary intake was also mentioned in our previous publication [18]. In brief, qualified dietitians taught patients how to fill in the record. To assure the record, the well-trained dietitians contacted with all patients and conducted the interviews by face-to-face, or by telephone. Next, the dietitians used the 24 h recall to confirm the data provided by patients using the common utensils in the household as the means. The nutrients were then analyzed using nutrients analysis software (e-Kitchen, Taichung, Taiwan) based on Taiwanese nutrition compositions as the nutrient database.

#### 2.2.4. Biochemical Values

We collected the 8-hour fasting and predialysis blood samples and sent to the Laboratory Department in Taipei Medical University Hospital for analyzing the biochemical parameters. The following parameters were collected by reviewing patient medical charts: total cholesterol, creatinine, and fasting blood glucose.

### 2.3. Measurements

#### 2.3.1. Homocysteine Measurements

Homocysteine was measured used in the enzymatic method. 8-hour fasting and predialysis blood samples were withdrawn and collected in EDTA blood collection tubes keep tube cold at 4°C and centrifuged within 1-2 hour, then plasma was assessed using the Roche Cobas c702 automatic analyzer (Rui An international Co. Ltd., Taipei, Taiwan) at the of the Laboratory Department in Taipei Medical University Hospital.

#### 2.3.2. Other Biochemical Measurements

The 8-hour fasting and predialysis blood samples were analyzed using standardized protocols for serum albumin, insulin, serum folate, serum vitamin B_12_, high-sensitivity C-reactive protein (hs-CRP), thyroid-stimulating hormone, and plasma homocysteine. The cutoff values for folate and B_12_ were 3 ng/mL and 250 pg/mL, respectively. Additionally, insulin resistance was measured by the homeostasis model assessment-estimated insulin resistance (HOMA-IR) calculated as (glucose [mg/dL] × insulin [*μ*U/mL)]/405 [19]. In the clinical assessment of nutritional status, we calculated the geriatric nutritional risk index (GNRI) as described previously, when GNRI < 91.2 exhibited a poorer nutritional status. Hypoalbuminemia and inflammation were defined as serum albumin less than 3.5 g/dL and hs-CRP higher than 0.5 mg/dL, respectively. Normalized protein nitrogen appearance (nPNA) ≥ 1.2 g/kg was appropriate protein intake in hemodialysis patients as suggested by the National Kidney Foundation [20].

#### 2.3.3. Anthropometry Measurements

Skeletal muscle mass (SMM) and body fat are measured by using the bioelectrical impedance analysis (InBody S10, Biospace, Seoul, Korea) after the hemodialysis session (sitting position). The eight surface electrodes are placed on the thumbs, middle fingers, and either side of the ankles of the patients using multiple operating frequencies of 1, 5, 50, 250, 500, and 1,000 kHz. Moreover, SMM was normalized for weight as SMM_Wt_ (%) and height as SMM_Ht2_ (kg/m^2^), which are indicators of muscle mass. Muscle quality (MQ) is an important determinant of muscle function, defined as muscle strength or power per unit of muscle mass and calculated as the ratio of hand grip strength (kg) to arm lean mass (kg) [21]. The handgrip strength (HGS) was measured before a hemodialysis session using grip strength dynamometer (Jamar, Sammons Preston, Bolingbrook, IL) with a precision of 0.5 kg. During the measurement, patients were asked to stand straight with arm and hands being neutrally hung beside the body and then use maximum effort to squeeze the dynamometer with nonfistula hand for at least 3 seconds, and each time was provided at least 10 seconds for recovery. Patient performed the handgrip for 3 times and the pick performance was noted as the final result.

### 2.4. Statistical Analyses

The sample of 138 patients with data of muscle mass and a sample of 87 patients with the data of muscle function were analyzed. The descriptive analyses were used to illustrate the mean ± standard deviation, percentage of social demographics, biochemical parameters, dietary intake, and patients' characteristics. Simple regression was used to identify the predictors of muscle mass and muscle function which were known as prognostic factors, statistical significance when p < 0.05. Multivariate linear regression models were performed to investigate the associations of Hcy and the muscle mass and muscle function. The variables showed the associations with muscle mass and function at p < 0.2 in the simple regression analyses which were kept in the multivariate models. Variables such as age, gender, and energy intake were also included in the analysis. The unstandardized regression coefficient (B), 95% confidence interval, and adjusted R square values were presented appropriately. Statistical analyses were performed using SAS software (ver. 9.4; SAS Institute Inc., Cary, NC, USA).

## 3. Results

### 3.1. Characteristics of Hemodialysis Patients

Demographics, anthropometrics, biochemical parameters, and clinical information are shown in Table 1. In 138 hemodialysis patients, 63.0% are men; 47.1 %, 51.5 %, and 43.5 % of the hemodialysis patients had DM, hypertension, and a history of CVD, respectively. The means of age and hemodialysis vintage were 73.1 ± 6.4 years and 4.5 ± 3.4 years, respectively. The plasma Hcy level was 19.7 ± 6.0 *μ*mol/L; 74.6 % of hemodialysis patients present in hyperhomocysteinemia (≥ 15 *μ*mol/L). The serum folate and vitamin B_12_ were 18.1 ± 4.5 ng/mL and 1416.8 ± 582.6 pg/mL, respectively. Hemodialysis patients were in a healthier and had good nutritional status, according to the GNRI 98.6 ± 7.1, serum albumin 3.9 ± 0.4 g/dL, and nPNA 1.3 ± 0.4 g/day, respectively (Table 1).

### 3.2. Associated Factors of Muscle Mass and Muscle Function

The SMM, SMM_Ht2_, and SMM_Wt_ were positively correlated with creatinine and height and were negatively correlated with age, gender, and body fat. HGS was positively associated with creatinine and height and was negatively correlated with gender. Muscle quality (MQ) was negatively correlated with body weight and BMI (Table 2).

The results stepwise regressions show that SMM was significant associations with energy intake (regression coefficient, B = < 0.01), BMI (B = 1.03), and body fat (B = -0.37) (adjusted R^2^ = 0.82), SMM_Ht2_ was significant associations with creatinine (B = 0.23) and body fat (B = -0.04) (adjusted R^2^ = 0.50), SMM_Wt_ was significant associations with Hcy (B = -0.03), creatinine (B = 0.13), and body fat (B = -0.57) (adjusted R^2^ = 0.97) (Table 3). In muscle function, HGS was significant associations with creatinine (B = 0.93) (adjusted R^2^ = 0.35); MQ was significant associations with creatinine (B = 0.45) and BMI (B = -0.58) (adjusted R^2^ = 0.18). Plasma Hcy level was the independent risk determinants of SMM_Wt_ in hemodialysis patients ≥ 65 years old (adjusted R^2^ = 0.97, p = 0.02).

After controlling the analysis subgroup of hyperhomocysteinemia hemodialysis patients, Hcy was significantly associated with SMM_Wt_ (B = -0.03, p = 0.027) (adjusted R^2^ = 0.99), HGS (B = -0.41, p = 0.058) (adjusted R^2^ = 0.35), and MQ (B = -0.18, p = 0.071) (adjusted R^2^ = 0.19) in hyperhomocysteinemia hemodialysis patients (Table 4).

## 4. Discussion

The current study found that Hcy was significantly inversely associated with muscle mass in hemodialysis patients aged 65 years and above. Besides, higher Hcy was marginally associated with muscle function decline in hyperhomocysteinemia hemodialysis patients aged 65 years and above. A previous longitudinal study has confirmed the association between higher homocysteine lower muscle strength in a general population aged 50 years or older [22]. Contributing factors such as age, low physical performance, and the presence of comorbidities were associated with muscle wasting [23].

The previous studies showed that elevated Hcy is associated with muscle function declined in the older adult [15, 16]. In addition to multiple hypotheses hyperhomocysteinemia impaired muscle function concluded by Veeranki and Tyagi [14], Hcy thiolactone reacts with proteins by a mechanism involving homocysteinylation of protein lysine residues in human serum and leads to protein damage and atrophy of skeletal muscle [24], the phenomenon called protein homocysteinylation [24]. Protein homocysteinylation is the posttranslational acylation of free amino groups (e.g., protein-N-homocysteinylation) or formation of a covalent–S–S–bond (protein-S-homocysteinylation) mediated by Hcy thiolactone [24], which potentially causes significant alterations in the protein function. A study reported that protein-N-homocysteinylation and protein-S-homocysteinylation were significantly higher in hemodialysis patients, and the significant association between plasma Hcy and protein-S-homocysteinylation was also found [25]. This indicated that hyperhomocysteinemia may cause the toxicity via the oxidative damage to proteins [26] and may result in muscle weakness and atrophy [24].

In our study, Kt/V was significantly associated with the lower SMM in hemodialysis patients. The negative association between Kt/V and muscle mass was also demonstrated in 34 Japanese hemodialysis patients [27]. The result indicated that patients with lower muscle mass may require a higher dialysis clearance. Therefore, muscle mass should be addressed while evaluating the hemodialysis adequacy.

The present study showed that dialysis vintage, creatinine, and BMI were independently associated with muscle mass and/or muscle function. The predialysis serum creatinine level will be proportional to dietary protein intake and the SMM that lower muscle mass may indicate the lower creatinine excretion [28]. The previous study showed that the hemodialysis vintage was negatively associated with the loss of SMM and muscle function. In addition, BMI was positively associated with SMM [27]. The possible reason for the negative correlation between BMI and MQ was supposed to be the calculation formula, BMI would be increase, and MQ would be decreased when skeletal muscle mass increases.

Our study shows that body fat was an independent determinant of muscle mass and muscle function among hemodialysis patients. A previous study was conducted using the computed tomography (CT) to take mid-thigh muscle cross-sectional area in a 36-year-old hemodialysis patient and an 80-year-old hemodialysis patient. Results showed that the fat accumulated in the striated muscle cells and intermuscular adipose tissue in older hemodialysis patients. Besides, the results also reveal that reduced muscle cross-sectional area, increased intermuscular adipose tissue, and/or intramuscular lipid were independently associated with aging and indices of maximal strength and physical performance [29].

Our study was with a number of strengths and limitations. The plasma homocysteine was analyzed by the international standard protocol in the hospital. We used the bioelectrical impedance analysis (BIA) as body composition measurement. The BIA is inexpensive, portable, and easy-to-use as compared with others devices. The measurement conducted by BIA might be minor differences as compared with other methods such as the dual-energy X-ray absorptiometry (DEXA), CT, and magnetic resonance imaging (MRI). However, the examination of total-body muscle mass using the BIA was highly correlated with estimation using the MRI in hemodialysis patients [30]. Finally, the basis of a cross-sectional design cannot provide the causal evidence between plasma Hcy and muscle mass. Therefore, the results must be interpreted with caution. Future cohort designs or control trials are suggested.

## 5. Conclusion

The current study revealed that hyperhomocysteinemia is high prevalence in hemodialysis patients. Elevated homocysteine level is associated with lower muscle mass in hemodialysis patients aged 65 years and above. Thus, the homocysteine-lowering therapy might have a positive impact on muscle mass. The longitudinal studies are suggested to provide a deeper understanding of the association. Randomized control trials are needed for evaluating the effectiveness of therapy.

## Figures and Tables

**Table 1:** Demographic, anthropometric, dietary, biochemical values, indicators of muscle mass, and muscle function in hemodialysis patients.

Variables	All (n = 138)
Demographics	
Age (years)	73.1 ± 6.4
Male	87 (63.0%)
Dialysis vintage (year)	4.5 ± 3.4
Interdialytic weight gain (%)	3.3 ± 1.2
eKt/V	1.6 ± 0.3
Diabetes mellitus	65 (47.1%)
Hypertension	71 (51.5%)
History of CVD	60 (43.5%)
Biochemical values	
Albumin (g/dL)	3.9 ± 0.4
Total cholesterol (mg/dL)	161.7 ± 34.8
Creatinine (mg/dL)	10.5 ± 1.8
FPG (mg/dL)	137.7 ± 63.2
Insulin (*μ*U/mL)	23.6 ± 22.4
HOMA-IR	9.1 ± 12.3
Homocysteine (*μ*mol/L)	19.7 ± 6.0
Hyperhomocysteinemia	103 (74.6%)
Folate (ng/mL)	18.1 ± 4.5
Vitamin B_12_ (pg/mL)	1416.8 ± 582.6
TSH (*μ*U/mL)	2.5 ± 2.1
hs-CRP (mg/dL)	0.6 ± 0.9
Anthropometrics	
Height (cm)	160.6 ± 7.9
Body weight (kg)	60.3 ± 9.7
BMI (kg/m^2^)	23.3 ± 3.0
Body fat (%)	29.9 ± 9.2
Dietary intake	
Energy (kcal/day)	1554.5 ± 494.5
Protein (g/day)	61.6 ± 23.9
Total fat (g/day)	61.9 ± 27.9
Meat and beans (serving/day)	5.1 ± 3.0
Vegetables (serving/day)	1.9 ± 1.5
Fruits (serving/day)	0.9 ± 1.0
Oil (serving/day)	3.7 ± 3.0
Folate (*μ*g/day)	435.8 ± 204.0
Vitamin B_2_ (mg/day)	1.5 ± 0.8
Vitamin B_6_ (mg/day)	2.1 ± 1.1
Vitamin B_12_ (*μ*g/day)	4.7 ± 3.1
Supplement	
Folic acid, n (%)	108 (78.3%)
B-complex, n (%)	96 (69.6%)
Muscle mass	
SMM (kg)	22.6 ± 4.5
SMM_Ht2_ (kg/m^2^)	8.7 ± 1.2
SMM_Wt_ (%)	37.7 ± 5.6
Muscle function ^a^	
Hand grip strength (kg)	21.1 ± 7.4
Arm lean mass (kg)	1.7 ± 0.5
Muscle quality	10.0 ± 3.0
Others	
nPNA (g/kg)	1.3 ± 0.4
GNRI	98.6 ± 7.1
MET (kcal/day)	753.8 ± 311.7

Values are expressed as mean ± standard deviation or number of patients (percentage).

^a^Sample of 87 patients with the data of muscle function was analyzed.

SD, standard deviation; CVD, cardiovascular disease; FPG, fasting plasma glucose; HOMA-IR, homoeostasis model assessment-estimated insulin resistance; TSH, thyroid-stimulating hormone; hs-CRP, high sensitivity C-reactive protein; BMI, body mass index; SMM, skeletal muscle mass; Ht, height; Wt, weight; nPNA, normalized protein nitrogen appearance; GNRI, geriatric nutritional risk index; MET, metabolic equivalent.

**Table 2:** Associated factors of muscle mass and muscle function via simple linear regression analyses.

	SMM		SMM_Ht2_		SMM_Wt_		Hand grip strength		Muscle quality	
Variables	B (95% CI)	*p*	B (95% CI)	*p*	B (95% CI)	*p*	B (95% CI)	*p*	B (95% CI)	*p*
Age (years)	-0.123 (-0.244 to -0.003)	0.045	-0.045 (-0.077 to -0.014)	0.005	-0.177 (-0.326 to -0.029)	0.020	-0.311 (-0.651 to 0.029)	0.072	-0.090 (-0.244 to 0.064)	0.243
Male, n (%)	-6.222 (-7.426 to -5.017)	< 0.001	-1.274 (-1.637 to -0.912)	< 0.001	-5.449 (-7.209 to -3.689)	< 0.001	-6.228 (-10.669 to -1.786)	0.007	0.966 (-1.180 to 3.113)	0.367
Dialysis vintage (year)	-0.001 (-0.230 to 0.229)	0.997	0.012 (-0.048 to 0.073)	0.688	0.170 (-0.113 to 0.453)	0.237	-0.179 (-0.829 to 0.472)	0.580	-0.013 (-0.301 to 0.275)	0.929
eKt/V	-5.448 (-7.742 to -3.153)	< 0.001	-1.094 (-1.719 to -0.470)	0.001	-2.657 (-5.691 to 0.378)	0.086	3.500 (-2.446 to 9.446)	0.240	2.188 (-0.378 to 4.754)	0.092
Albumin (g/dL)	1.441 (-0.354 to 3.237)	0.115	0.334 (-0.139 to 0.808)	0.165	0.070 (-2.175 to 2.314)	0.951	3.270 (-3.903 to 10.443)	0.361	0.248 (-2.950 to 3.447)	0.876
Creatinine (mg/dL)	1.158 (0.784 to 1.531)	< 0.001	0.312 (0.215 to 0.410)	< 0.001	0.513 (-0.003 to 1.030)	0.051	1.161 (0.065 to 2.257)	0.039	-0.042 (-0.556 to 0.472)	0.869
FPG (mg/dL)	-0.005 (-0.017 to 0.007)	0.404	-0.002 (-0.005 to 0.001)	0.231	-0.008 (-0.023 to 0.007)	0.291	-0.015 (-0.083 to 0.053)	0.663	-0.005 (-0.035 to 0.025)	0.725
Insulin (*μ*U/mL)	-0.005 (-0.039 to 0.030)	0.779	-0.003 (-0.012 to 0.007)	0.587	-0.045 (-0.087 to -0.003)	0.037	-0.066 (-0.168 to 0.036)	0.196	-0.002 (-0.048 to 0.044)	0.925
HOMA-IR	-0.019 (-0.082 to 0.044)	0.552	-0.007 (-0.023 to 0.010)	0.413	-0.072 (-0.149 to 0.005)	0.066	-0.198 (-0.481 to 0.084)	0.162	-0.010 (-0.138 to 0.118)	0.872
Homocysteine (*μ*mol/L)	-0.008 (-0.138 to 0.122)	0.908	-0.009 (-0.043 to 0.025)	0.592	0.060 (-0.101 to 0.221)	0.463	0.385 (-0.080 to 0.851)	0.101	-0.025 (-0.238 to 0.188)	0.816
Folate (ng/mL)	0.040 (-0.131 to 0.211)	0.648	0.011 (-0.034 to 0.056)	0.635	-0.001 (-0.213 to 0.211)	0.991	-0.164 (-0.538 to 0.209)	0.378	0.049 (-0.116 to 0.215)	0.548
Vitamin B_12_ (pg/mL)	0.001 (-0.001 to 0.002)	0.594	0.000 (-0.001 to 0.001)	0.809	-0.001 (-0.002 to 0.001)	0.376	-0.001 (-0.005 to 0.003)	0.739	0.001(-0.002 to 0.002)	0.853
Energy (kcal/day)	0.003 (0.001 to 0.004)	0.001	0.0005 (0.000 to 0.001)	0.033	0.001 (-0.001 to 0.003)	0.371	0.003 (-0.001 to 0.008)	0.087	0.001(-0.002 to 0.002)	0.914
Height (cm)	0.454 (0.393 to 0.515)	< 0.001	0.067 (0.044 to 0.090)	< 0.001	0.278 (0.166 to 0.391)	< 0.001	0.392 (0.141 to 0.643)	0.003	-0.042 (-0.166 to 0.083)	0.504
Body weight (kg)	0.316 (0.257 to 0.375)	< 0.001	0.067 (0.050 to 0.085)	< 0.001	-0.100 (-0.198 to -0.003)	0.044	0.193 (-0.050 to 0.436)	0.116	-0.127 (-0.229 to -0.025)	0.016
BMI (kg/m^2^)	0.381 (0.128 to 0.634)	0.004	0.146 (0.082 to 0.210)	< 0.001	-0.982 (-1.259 to -0.706)	< 0.001	-0.135 (-0.897 to 0.627)	0.721	-0.373 (-0.684 to -0.062)	0.020
Body fat (%)	-0.244 (-0.317 to -0.172)	< 0.001	-0.065 (-0.084 to -0.046)	< 0.001	-0.591 (-0.612 to -0.570)	< 0.001	-0.171 (-0.441 to 0.100)	0.209	-0.019 (-0.141 to 0.103)	0.752
MET (kcal/day)	0.004 (0.002 to 0.007)	< 0.001	0.0015 (0.001 to 0.002)	0.004	0.003 (-0.001 to 0.006)	0.052	0.001 (-0.008 to 0.011)	0.791	-0.001 (-0.005 to 0.003)	0.660

FPG, fasting plasma glucose; HOMA-IR, homeostasis model assessment-estimated insulin resistance; BMI, body mass index; MET, metabolic equivalent; SMM, skeletal muscle mass; Ht, height; Wt, weight.

**Table 3:** Stepwise regression for determining muscle mass and muscle function in hemodialysis patients.

Variables	B (95% CI) ^a^	*p*	adjusted R^2^
SMM			0.82
Kt/V	-1.13 (-2.28 to 0.03)	0.056	
Energy (kcal/day)	0.0008 (0.0001 to 0.0015)	0.034	
BMI (kg/m^2^)	1.03 (0.88 to 1.19)	< 0.001	
Body fat (%)	-0.37 (-0.42 to -0.31)	< 0.001	
MET (kcal/day)	0.0009 (-0.0002 to 0.0020)	0.102	
SMM_Ht2_			0.50
Homocysteine (*μ*mol/L)	-0.02 (-0.05 to 0.01)	0.060	
Creatinine (mg/dL)	0.23 (0.14 to 0.31)	< 0.001	
Body fat (%)	-0.04 (-0.06 to -0.03)	< 0.001	
SMM_Wt_			0.97
Homocysteine (*μ*mol/L)	-0.03 (-0.06 to -0.01)	0.020	
Creatinine (mg/dL)	0.13 (0.03 to 0.22)	0.009	
Body fat (%)	-0.57 (-0.59 to -0.55)	< 0.001	
Hand grip strength			0.35
Creatinine (mg/dL)	0.93 (0.18 to 1.68)	0.015	
Energy (kcal/day)	0.0023 (-0.0004 to 0.0050)	0.092	
Body fat (%)	-0.14 (-0.31 to 0.03)	0.101	
Muscle quality^2^			0.18
Dialysis vintage (year)	-0.15 (-0.35 to 0.05)	0.145	
Kt/V	1.91 (-0.08 to 3.91)	0.060	
Creatinine (mg/dL)	0.45 (0.06 to 0.83)	0.023	
BMI (kg/m^2^)	-0.58 (-0.88 to -0.29)	< 0.001	
Body fat (%)	0.07 (-0.04 to 0.18)	0.190	

^a^The significance levels of any potential factor for entry (SLE) and for stay (SLS) in the stepwise variable selection were set to 0.2. Age and gender were adjusted. Variables retained in the model are presented in the table.

BMI, body mass index; MET, metabolic equivalent; SMM, skeletal muscle mass; Ht, height; Wt, weight.

**Table 4:** Stepwise regression analyses for determining muscle mass and muscle function in hemodialysis patients with hyperhomocysteinemia.

Variables	B (95% CI) ^a^	*p*	adjusted R^2^
SMM			0.84
Creatinine (mg/dL)	0.23 (0.01 to 0.45)	0.042	
BMI (kg/m^2^)	1.02 (0.86 to 1.18)	< 0.001	
Body fat (%)	-0.36 (-0.41 to -0.30)	< 0.001	
SMM_Ht2_			0.47
Creatinine (mg/dL)	0.18 (0.07 to 0.29)	0.002	
Body fat (%)	-0.04 (-0.06 to -0.02)	< 0.001	
MET (kcal/day)	0.0006 (-0.0001 to 0.0012)	0.084	
SMM_Wt_			0.99
Homocysteine (*μ*mol/L)	-0.03 (-0.05 to -0.01)	0.027	
Creatinine (mg/dL)	0.17 (0.09 to 0.25)	< 0.001	
Body fat (%)	-0.55 (-0.56 to -0.53)	< 0.001	
Hand grip strength			0.35
Homocysteine (*μ*mol/L)	-0.41 (-0.83 to 0.01)	0.058	
Dialysis vintage (year)	-0.40 (-0.93 to 0.14)	0.143	
eKt/V	6.65 (1.39 to 11.91)	0.014	
Creatinine (mg/dL)	1.34 (0.37 to 2.32)	0.008	
Energy (kcal/day)	0.0042 (0.0008 to 0.0075)	0.015	
Body fat (%)	-0.19 (-0.40 to 0.02)	0.079	
Muscle quality^2^			0.19
Homocysteine (*μ*mol/L)	-0.18 (-0.37 to 0.02)	0.071	
Dialysis vintage (year)	-0.22 (-0.46 to 0.02)	0.068	
eKt/V	3.14 (0.76 to 5.53)	0.011	
Creatinine (mg/dL)	0.54 (0.08 to 1.01)	0.023	
Energy (kcal/day)	0.0015 (0.00005 to 0.0031)	0.045	
BMI (kg/m^2^)	-0.47 (-0.73 to -0.21)	< 0.001	

^a^The significance levels of any potential factor for entry (SLE) and for stay (SLS) in the stepwise variable selection were set to 0.2. Age and gender were adjusted. Variables retained in the model are presented in the table.

BMI, body mass index; MET, metabolic equivalent; SMM, skeletal muscle mass; Ht, height; Wt, weight.

## Data Availability

Data is available upon request to corresponding author of this article.
